# A case of membranous nephropathy diagnosed with lupus nephritis 11 years after onset

**DOI:** 10.1007/s13730-019-00412-5

**Published:** 2019-08-09

**Authors:** Takehisa Yamada, Fumiaki Itagaki, Sae Aratani, Sayuri Kawasaki, Kousuke Terada, Koji Mugishima, Tetsuya Kashiwagi, Akira Shimizu, Shuichi Tsuruoka

**Affiliations:** 1 Department of Nephrology, Nippon Medical School Chiba Hokusoh Hospital, 1715, Kamagari, Inzai, Chiba 270-1694 Japan; 2 Department of Nephrology, Nippon Medical School Musashi Kosugi Hospital, 1-396, Kosugimachi, Nakahara-ku, Kawasaki, Kanagawa 211-8533 Japan; 3 Department of Nephrology, Nippon Medical School, 1-1-5, Sendagi, Bunkyo-ku, Tokyo, 113-8603 Japan; 4 Department of Analytic Human Pathology, Nippon Medical School, 1-1-5, Sendagi, Bunkyo-ku, Tokyo, 113-8603 Japan

**Keywords:** Membranous lupus nephritis, Lupus-like membranous nephropathy, Tubuloreticular inclusion, IgG subclass

## Abstract

A 34-year-old female patient presented to our hospital with lower extremity edema and proteinuria during pregnancy. Renal biopsy was performed and the patient was diagnosed with nephrotic syndrome due to lupus-like membranous nephropathy. This diagnosis was reached upon as laboratory findings upon admission, wherein both anti-nuclear and anti-double-stranded DNA antibodies revealed negative, did not fulfill the criteria for systemic lupus erythematosus (SLE) proposed by the American College of Rheumatology (ACR) and the patient did not reveal any typical physical manifestations of SLE. Methylprednisolone pulse therapy was started followed by oral administration of prednisolone. Urinary protein excretion diminished after 1 year of treatment. Eleven years later, the same patient was admitted to our hospital again with relapse of nephrotic syndrome. Laboratory findings upon second admission, wherein both anti-nuclear and anti-double-stranded DNA antibodies revealed positive, fulfilled the ACR criteria. Renal biopsy was performed again, resulting in a diagnosis of lupus nephritis. Steroid therapy combined with administration of mycophenolate mofetil led to an incomplete remission. Immunofluorescence studies confirmed the presence of IgG, IgM, C3, and C1q in renal biopsy specimens both at first and second admissions. Furthermore, immunofluorescence studies confirmed the presence of IgG1–4 in the first biopsy and tubuloreticular inclusions (TRIs) were revealed using electron microscopy. The present case represents the possibility that characteristic pathological findings of lupus nephritis, including TRIs, can reveal themselves before a diagnosis of SLE.

## Introduction

There has been a growing body of reports regarding membranous nephropathy (MN) cases that present like lupus nephritis [1–7] without fulfilling the classification criteria for systemic lupus erythematosus (SLE) proposed by the American College of Rheumatology (ACR) [8–10]. The importance of pathological findings from renal biopsy specimens has been highlighted by the recently defined classification criteria for SLE, proposed by the Systemic Lupus International Collaborating Clinics (SLICC) group in 2012 [11]. These new classification proposals suggest that a diagnosis for SLE can be reached not only in patients who fulfill at least four criteria, including at least one clinical and one immunological criteria, but also in patients with biopsy-proven lupus nephritis combined with the presence of anti-nuclear antibodies (ANA) or anti-double-stranded DNA (anti-dsDNA) antibodies [11]. Until this new proposal was made, cases which did not satisfy at least four criteria were tended to be excluded from a possibility of SLE, even though they had shown characteristic pathological findings of lupus nephritis.

Recently, we encountered a female patient who had SLE with lupus nephritis. At first, the patient presented to our hospital with nephrotic syndrome. According to the ACR criteria [10], which were formerly conventionally used at first admission, the patient was excluded from a possibility of SLE, despite pathological findings of lupus-like MN, because they tested negative for both ANA and anti-dsDNA antibodies. Eleven years later, the same patient was admitted to our hospital again due to relapse of nephrotic syndrome. Laboratory findings upon second admission, wherein both ANA and anti-dsDNA antibodies revealed positive, fulfilled both ACR and SLICC criteria [10, 11].

## Case report

**Fig. 1:**
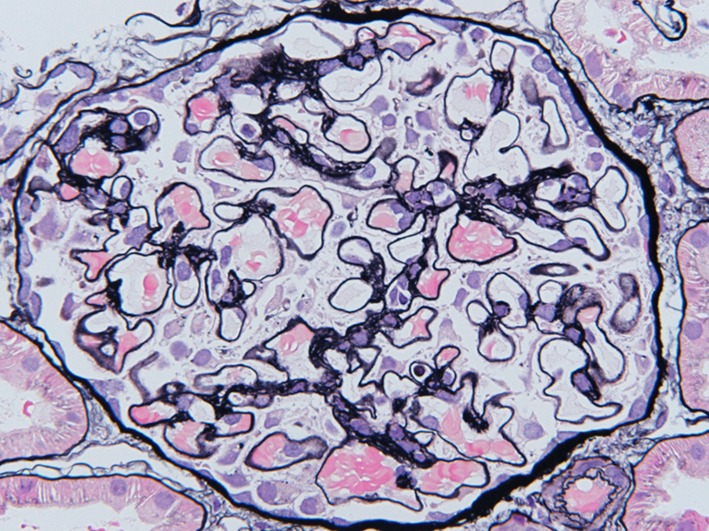
Pathological findings in renal biopsy during first admission. Light microscopy revealed mild thickening of glomerular basement membrane with stippling and spike formation (PASM staining, × 400)

**Fig. 2:**
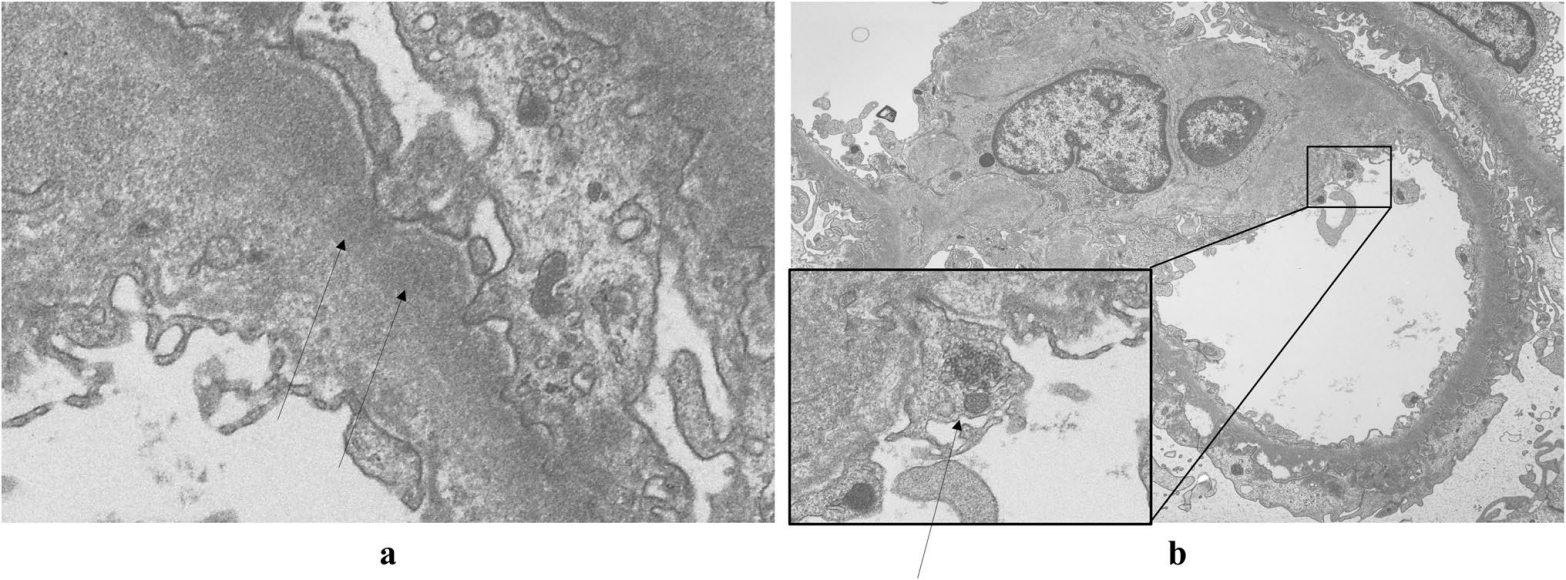
Electron microscopy revealed slight subepithelial deposits (arrows) (**a**). Notably, a magnified view of the square demonstrated tubuloreticular inclusion (arrow) in the endothelial cell (**b**)

**Fig. 3:**
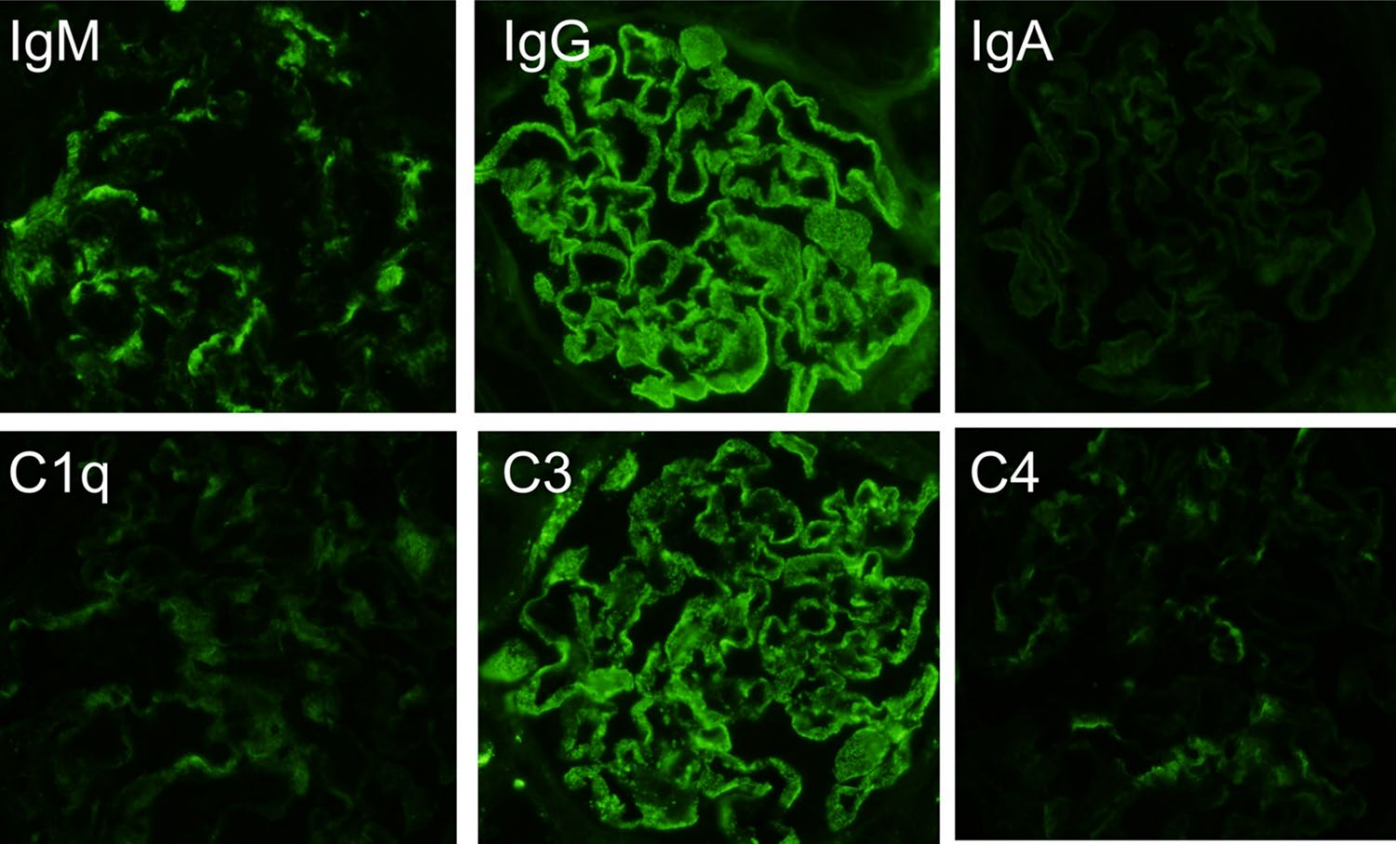
Immunofluorescence studies revealed granular deposits of IgG, IgM, C3, and C1q, with only slight deposits of C4 and IgA in the glomeruli

**Fig. 4:**
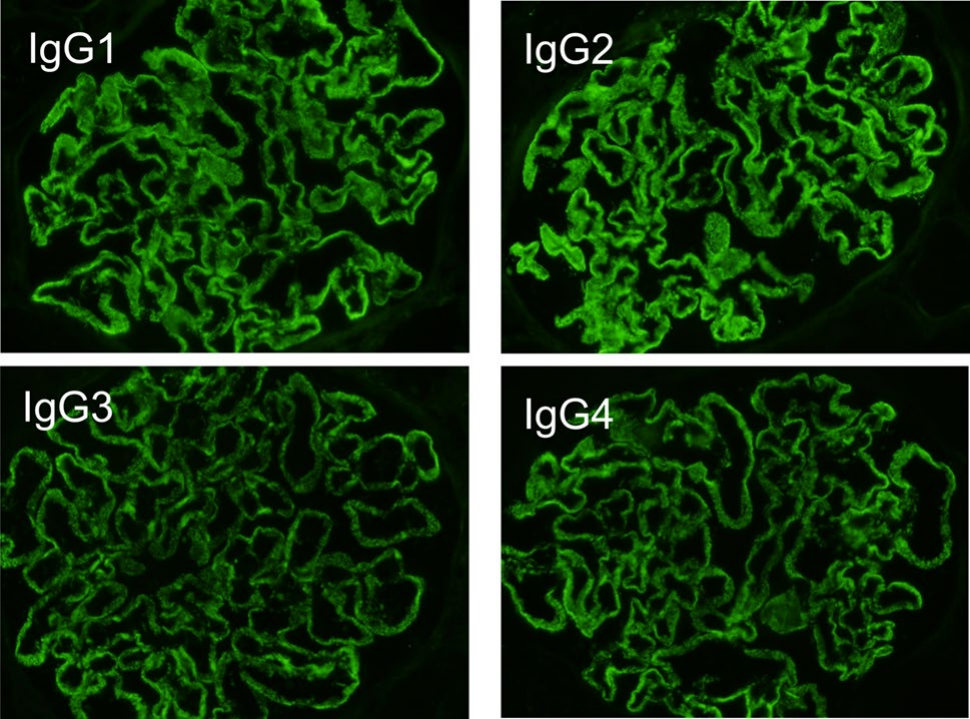
Immunofluorescence studies of IgG subclasses revealed positive stains for all four (IgG1–4)

A 34-year-old woman presented to our hospital with edema of the lower extremities during the eighth. week of pregnancy. She had had neither reproductive nor obstetric history before. She had neither skin rash nor arthralgia. She had neither photosensitivity nor oral ulcers. Laboratory tests conducted upon admission revealed hypoalbuminemia, proteinuria, and hyperlipidemia. Both ANA and anti-dsDNA antibody test yielded negative results. Levels of CH50, C3, and C4 were all normal. The levels of hepatitis B surface-antigen and hepatitis C virus antibodies were also normal (Table 1). There were no symptoms of malignancy. Percutaneous renal biopsy yielded characteristic findings of MN, which were revealed using light microscopy (Fig. 1). Pathological features, including slight subepithelial deposits and the presence of tubuloreticular inclusions in endothelial cells, were revealed using electron microscopy (Fig. 2a, b). Although immunofluorescence studies yielded positive stains for IgG (including all four subclasses, IgG1–4), IgM, C3 and C1q (Figs. 3, 4), the patient was diagnosed with lupus-like MN because laboratory findings upon admission did not fulfill the ACR criteria [10] and there were no typical physical manifestations of SLE. Methylprednisolone pulse therapy followed by oral administration of prednisolone was commenced, resulting in complete remission after 1 year. She delivered normally without any complication at 37th week of her pregnancy. After first remission, the patient was transferred to her primary care doctor. Laboratory findings for the patient were therefore not observed until second admission.

**Table 1:** Laboratory findings at first admission

Urinalysis		BC		IgA	179 mg/dL
Pro.	(3 +)	T.Bil	0.1 mg/dL	IgM	183 mg/dL
Glu.	(−)	AST	13 IU/L	CH50	38.0 U/dL
Urobili.	(±)	ALT	10 IU/L	C3	130 mg/dL
Bil.	(−)	UA	3.0 mg/dL	C4	33.2 mg/dL
Ket.	(−)	LDH	130 IU/L	Anti-nuclear-Ab	< × 40
Occult blood	(1 +)	P	3.4 g/dL	Anti-dsDNAIgG	2.4 IU/mL
Sed.		Alb	1.4 g/dL	PR3-ANCA	< 1.3 U/mL
RBC	50–99/HPF	CK	29 IU/L	MPO-ANCA	< 1.3 U/mL
WBC	5–9/HPF	BUN	5.1 mg/dL	Anti-GBM-Ab	< 10 EU
Casts	50–99/HPF	Cr	0.59 mg/dL	HBs-Ag	(−)
CBC		Na	139 mEq/dL	HCV-Ab	(−)
WBC	7870/mL	K	3.8 mEq/dL	TPHA	(−)
RBC	296 × 10^4^/mL	Cl	108 mEq/dL	RPR	(−)
Hb	9.3 g/dL	Glu.	75 mg/dL	Anti-SSA	(−)
Hct	26.3%	T.chol	547 mg/dL	Anti-SSB	(−)
MCV	78.2%	TG	172 mg/dL	Anti-CL-β2GP	< 1.3 U/mL
MCH	24.7 pg	Serology		Anti-cardio-IgG	2 U/mL
MCHC	31.5%	CRP	< 0.05 mg/dL	Urinary protein excretion	7.5 g/day
PLT	20.9 × 10^4^/mL	IgG	492 mg/dL	Selectivity index	0.117

Abnormal data have been underlined

*Anti*-*nuclear*-*Ab* antinuclear antibodies, *Anti*-*dsDNAIgG* anti-double stranded deoxyribonucleic acid-immunoglobulin G antibodies, *PR3*-*ANCA* proteinase3-antineutrophil cytoplasmic antibodies, *MPO*-*ANCA* myeloperoxidase-antineutrophil cytoplasmic antibodies, *Anti*-*GBM*-*Ab* anti-glomerular basement membrane antibodies, *Anti*-*CL*-*β2GP* anti-cardiolipin beta2 glycoprotein 1 antibodies, *TPHA* treponema pallidum hemagglutination test, *RPR* rapid plasma regain card agglutination test, *SS* Sjögren’s syndrome

**Fig. 5:**
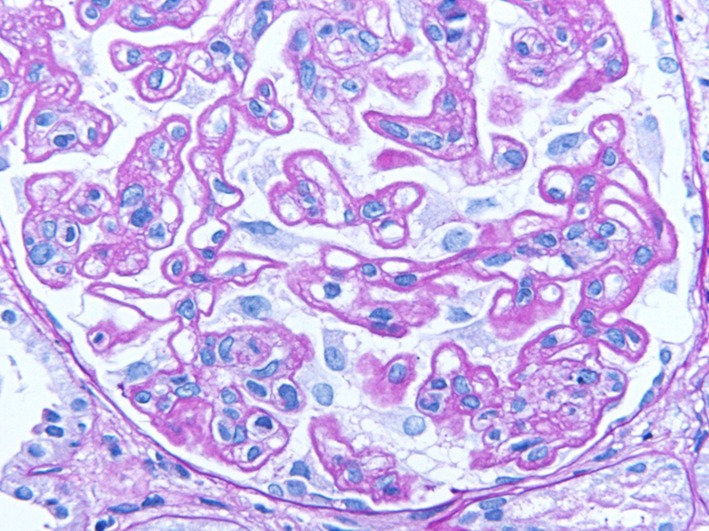
Pathological findings in renal biopsy during second admission. Double contour and hypercellularity in mesangial lesions represent a pattern of membranoproliferative glomerulonephritis (PAS staining, × 400)

**Fig. 6:**
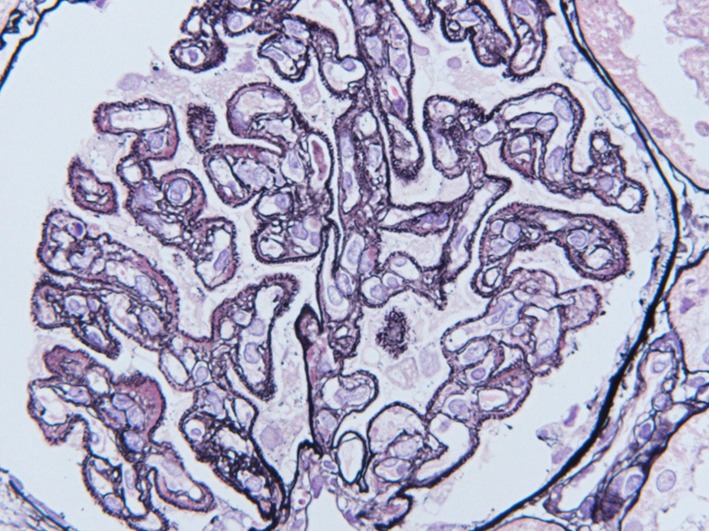
PASM staining revealed subepithelial deposits and spike formation (PASM staining, × 400)

**Fig. 7:**
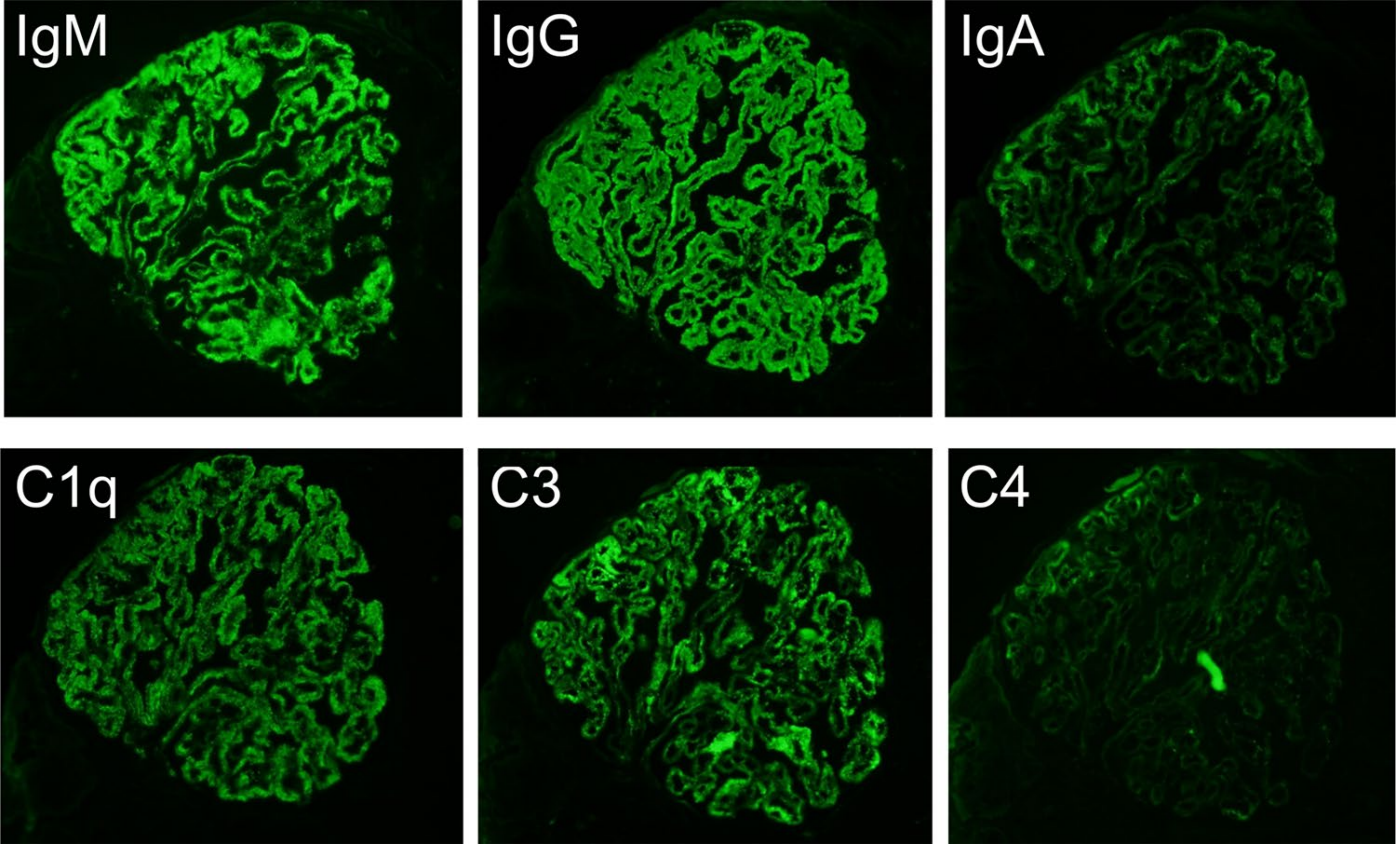
Immunofluorescence studies revealed positive mesangial and peripheral stains for IgG, IgM, IgA, C3, C1q, and C4

Eleven years later, the same patient presented to our hospital with edema of lower extremities and self-reported abdominal distension. She had not yet experienced menopause. Tests revealed hypoalbuminemia, proteinuria, and hyperlipidemia. The value of lupus anticoagulant was normal. Serological tests yielded positive results for both ANA and anti-dsDNA antibodies. Laboratory findings at second admission suggested leukocytopenia and hypocomplementemia (Table 2). These clinical findings fulfilled the ACR and the SLICC criteria [10, 11]. In addition to subepithelial spikes and deposits, hypercellularity in mesangial and endocapillary lesions were demonstrated in pathological findings from renal biopsy specimens upon second admission (Figs. 5, 6). These findings suggested membranoproliferative glomerulonephritis with positive stains for IgG, IgM, IgA, C3, and C1q (Fig. 7), which were compatible with class IV + V lupus nephritis. We could not obtain enough samples for an electron microscopic study at second renal biopsy. Methylprednisolone pulse therapy followed by oral administration of prednisolone combined with mycophenolate mofetil achieved incomplete remission after 6 months. The patient is currently under observation at our outpatient clinic.

**Table 2:** Laboratory findings at second admission

Urinalysis		BC		Serology	
Pro.	(4 +)	T.Bil	0.2 mg/dL	CRP	< 0.05 mg/dL
Glu.	(−)	AST	28 IU/L	IgG	649 mg/dL
Urobili.	(±)	ALT	12 IU/L	IgA	189 U/dL
Bil.	(−)	γ-GTP	10 IU/L	IgM	170 mg/dL
Ket.	(−)	ALP	99 IU/L	CH50	5 U/dL
Occult blood	(2 +)	LDH	247 IU/L	C3	3.6 mg/dL
Sed.		TP	3.5 g/dL	C4	34 mg/dL
RBC	5–9/HPF	Alb	1.3 g/dL	Anti-nuclear-Ab	× 1280 (homogeneous)
WBC	5–9/HPF	CK	135 IU/dL	Anti-dsDNAIgG	> 380 IU/mL
Casts	100–999/HPF	BUN	7.3 mg/dL	PR3-ANCA	< 0.5 U/mL
CBC		Cr	0.65 mg/dL	MPO-ANCA	< 0.5 U/mL
WBC	2790/mL	Na	144 mEq/dL	Anti-GBM-Ab	< 0.5 U/mL
RBC	406 × 10^4^/mL	K	3.0 mEq/dL	Anti-CL-β2GP	< 1.3 U/mL
Hb	9.1 g/dL	Cl	108 mEq/dL	Anti-cardio-IgG	7 U/mL
Hct	30.2%	Ca	7.2 mg/dL	HBs-Ag	(−)
MCV	74.4%	P	4.3 mg/dL	HCV-Ab	(−)
MCH	22.4 pg	T.chol	429 mg/dL	TPHA	(−)
MCHC	30.1%	TG	80 mg/dL	RPR	(−)
PLT	23.9 × 10^4^/mL	LDL-chol	399 mg/dL	Lupus anticoagulant	4.5 s
				Urinary protein excretion	16.6 g/day

Abnormal data have been underlined

*Anti*-*nuclear*-*Ab* antinuclear antibodies, *Anti*-*dsDNAIgG* anti-double stranded deoxyribonucleic acid-immunoglobulin G antibodies, *PR3*-*ANCA* proteinase3-antineutrophil cytoplasmic antibodies, *MPO*-*ANCA* myeloperoxidase-antineutrophil cytoplasmic antibodies, *Anti*-*GBM*-*Ab* anti-glomerular basement membrane antibodies, *Anti*-*CL-β2GP* anti-cardiolipin beta2 glycoprotein 1 antibodies, *TPHA* treponema pallidum hemagglutination test, *RPR* rapid plasma regain card agglutination test

## Discussion

The clinical criteria for SLE have been evolving ever since the ACR proposed the preliminary criteria [8] in 1971, the revised versions of which were proposed in 1982 and 1997 [9, 10]. Currently, the most widely used classification criteria for SLE are those revised and validated by SLICC [11]. According to their suggestions, at least four criteria,—including at least one clinical criterion and one immunological criteria—are necessary for the diagnosis of SLE. Importantly, however, SLICC criteria also suggest that a diagnosis of SLE can be made based on biopsy-proven lupus nephritis combined with the presence of ANA or anti-dsDNA antibodies [11]. Compared with the ACR criteria, the SLICC criteria highlight the importance of renal pathological findings in the process of diagnosing SLE, resulting in improved sensitivity compared with the ACR criteria [12].

Doi et al. have reported that there are differences in the distribution of IgG subclasses that are deposited in renal tissue between primary MN and lupus nephritis [13]. In their report, IgG4 was the predominant subclass detected in primary MN with granular deposits detected along the capillary walls of glomeruli; neither IgG2 nor IgG3 deposition was identified in patients with primary MN. Conversely, all IgG subclasses were detected in samples from lupus nephritis patients, with the prevalence of IgG1 and IgG3 [13].

The present case demonstrated characteristic findings of lupus-like MN, in which all IgG subclasses were detected by immunofluorescence studies (Fig. 4), although laboratory tests were negative for both ANA and anti-dsDNA antibodies upon first admission (Table 1). During first admission at which time SLICC criteria [11] had not yet been published, laboratory findings did not satisfy the current ACR criteria [10]. Furthermore, retrospectively, those laboratory findings also failed to satisfy the SLICC criteria [11], despite lupus-like features in the renal biopsy specimens (Figs. 2, 3), because test for both ANA and anti-dsDNA antibodies yielded negative results (Table 1). Eleven years later, serum titers of ANA and anti-dsDNA antibodies had increased, resulting in tests yielding positive results upon second admission (Table 2). Assessment of renal biopsy specimens at second admission demonstrated membranoproliferative glomerulonephritis via light microscopy and positive stains for IgG, IgM, IgA, C3, and C1q in immunofluorescence studies, compatible with class IV + V lupus nephritis (Figs. 5, 6, 7). These laboratory and pathological findings fulfilled SLICC criteria [11]. Although tests for both ANA and anti-dsDNA antibodies yielded negative results, immunofluorescence studies revealed positive stains for IgG, IgM, C3, and C1q combined with all positive stains for IgG subclasses in the first biopsy (Figs. 3, 4). Therefore, there remained the possibility that our patient would progress to SLE later—as suggested by lupus-like immunofluorescence findings.

Cases of lupus-like MN without a diagnosis of SLE have been reported [2–7] recently, beginning with Simenhoff and Merrill suggesting that lupus nephritis may present only as a renal manifestation, without any other manifestations of SLE [1]. In fact, some patients who have lupus-like pathological features in renal biopsy specimens, without a diagnosis of SLE, subsequently fulfill the criteria for SLE years later [2–7, 14]. The literature reviews have revealed that the average age of such patients is 24.7 years and that 25% develop SLE after a mean followup period of 5 years [14]; our patient was older and the time to develop SLE was longer, than those reported previously [2–7]. In such cases, it is necessary for physicians to continue to observe autoantibody levels even when the patients were excluded from a possibility of SLE during initial treatment.

In addition to IgG subclass stains, tubuloreticular inclusion can also play an important role in distinguishing secondary MN from primary [15]. Gianviti et al. have reported delayed onset of SLE in pediatric patients with lupus-like immunofluorescence patterns and cytoplasmic tubuloreticular inclusions in renal biopsies [7]. In the present case, observation of pathological features with an electron microscope revealed tubuloreticular inclusions in the endothelial cells in the first biopsy (Fig. 2b). Taking this together with the immunofluorescence findings (Figs. 3, 4), we conclude that the pathological features of MN in the first biopsy represented an early phase of membranous lupus nephropathy.

With regard to the effects of her pregnancy on SLE, it is well known that early phase of SLE is likely to be associated with pregnancy. It is also reported that risk of the disease exacerbation and pregnancy complications increase in patients with lupus nephritis [16–18]. We speculated that pregnancy might trigger off not only our patient’s lupus-like MN, but also the seroconversion of ANA and anti-dsDNA antibodies at second admission, while she delivered normally without any complication at 37th week of her pregnancy. It is reported that serum complement levels increase during pregnancy [19], while our patient’s serum complement levels were normal at first admission. The possibility was that the characteristic finding of low serum complement levels in SLE might be masked by her pregnancy. Although immunofluorescence for IgG subclasses revealed all positive stains for IgG1–4 and tubuloreticular inclusions were demonstrated in an electron microscopy, we diagnosed our patient with lupus-like MN, not lupus nephritis at first admission. It was because that the possibility of pregnancy-induced effect on autoimmune system in our patient could not be excluded and that there were no typical physical manifestations of SLE at first admission.

In conclusion, we have reported on a case of MN diagnosed with lupus nephritis 11 years after onset. Although the patient could not be diagnosed with SLE at first admission, lupus-like pathological findings were already observable in renal biopsy specimens. Immunofluorescence studies and the presence of tubuloreticular inclusions helped to differentiate lupus-like MN from idiopathic MN. In such cases, it is necessary for physicians to carefully observe whether the classification criteria for SLE are fulfilled after the remission of symptoms following initial treatment. The classification criteria proposed by SLICC [11] are therefore helpful for physicians to predict the chance of progression into SLE of patients who are excluded from a possibility of SLE but present with lupus-like nephropathy.

## References

[1] Simenhoff ML, Merrill JP (1964). The spectrum of lupus nephritis. Nephron.

[2] Kallen RJ, Lee SK, Aronson AJ, Spargo BH (1977). Idiopathic membranous glomerulonephropathy preceding the emergence of systemic lupus erythematosus in two children. J Pediatr.

[3] Caims SA, Corbett CL, Lawler W, Mallick NP, Acheson EJ, Dosa S, Williams DG (1979). The delayed appearance of an antinuclear factor and the diagnosis of systemic lupus erythematosus in glomerulonephritis. Postgrad Med J.

[4] Shearn MA, Hopper J, Biava CG (1980). Membranous lupus nephropathy initially seen as an idiopathic membranous nephropathy. Arch Intern Med.

[5] Adu D, Williams DG, Taube D, Vilches AR, Turner DR, Cameron JS, Ogg CS (1983). Late onset systemic lupus erythematosus and lupus-like disease in patients with apparent idiopathic glomerulonephritis. Q J Med.

[6] Wen YK, Chen ML (2010). Clinicopathological study of originally non-lupus “full-house” nephropathy. Renal Fail.

[7] Gianviti A, Barsotti P, Barbera V, Faraggiana T, Rizzoni G (1999). Delayed onset of systemic lupus erythematosus in patients with “full-house” nephropathy. Pediatr Nephrol.

[8] Cohen AS, Reynolds WE, Franklin EC, Kulka JP, Ropes MW, Shulman LE, Wallace SL (1971). Preliminary criteria for the classification of systemic lupus erythematosus. Bull Rheum Dis.

[9] Tan EM, Cohen AS, Fries JF, Masi AT, McShane DJ, Rothfield NF, Schaller JG, Talal N, Winchester RJ (1982). The 1982 revised criteria for the classification of systemic lupus erythematosus. Arthritis Rhuematol.

[10] Hochberg MC (1997). Updating the American College of Rheumatology revised criteria for the classification of systemic lupus erythematosus. Arthritis Rhuematol.

[11] Petri M, Orbai AM, Alarcón GS, Gordon C, Merrill JT, Fortin PR, Bruce IN, Isenberg D, Wallace DJ, Nived O, Sturfelt G, Ramsey-Goldman R, Bae SC, Hanly JG, Sánchez-Guerrero J, Clarke A, Aranow C, Manzi S, Urowitz M, Gladman D, Kalunian K, Costner M, Werth VP, Zoma A, Bernatsky S, Ruiz-Irastorza G, Khamashta MA, Jacobsen S, Buyon JP, Maddison P, Dooley MA, van Vollenhoven RF, Ginzler E, Stoll T, Peschken C, Jorizzo JL, Callen JP, Lim SS, Fessler BJ, Inanc M, Kamen DL, Rahman A, Steinsson K, Franks AG, Sigler L, Hameed S, Fang H, Pham N, Brey R, Weisman MH, McGwin G, Magder LS (2012). Derivation and validation of the Systemic Lupus International Collaborating Clinics classification criteria for systemic lupus erythematosus. Arthritis Rheumatol.

[12] Rijnink EC, Teng YKO, Kraaij T, Dekkers OM, Bruijn JA, Bajema IM (2018). Validation of the Systemic Lupus International Collaborating Clinics classification criteria in a cohort of patients with full house glomerular deposits. Kidney Int.

[13] Doi T, Mayumi M, Kanatsu K, Suehiro F, Hamashima Y (1984). Distribution of IgG subclasses in membranous nephropathy. Clin Exp Immunol.

[14] Sam R, Joshi A, James S, Jen KY, Amani F, Hart P, Schwartz MM (2015). Lupus-like membranous nephropathy: is it true or not?. Clin Exp Nephrol.

[15] Jeannette JC, Iskandar SS, Dalldorf FG (1983). Pathological differentiation between lupus and nonlupus membranous glomerulopathy. Kidney Int.

[16] Le Thi Huong D, Wechsler B, Piette JC, Bletry O, Godeau P (1994). Pregnancy and its outcome in systemic lupus erythematosus. Q J Med.

[17] Moroni G, Quaglini S, Banfi G, Caloni M, Finazzi S, Ambroso G, Como G, Ponticelli C (2002). Pregnancy in lupus nephritis. Am J Kidney Dis.

[18] Smyth A, Oliveira GH, Lahr BD, Bailey KR, Norby SM, Garovic VD (2010). A systematic review and meta-analysis of pregnancy outcomes in patients with systemic lupus erythematosus and lupus nephritis. Clin J Am Soc Nephrol.

[19] Johnson U, Gustavii B (1987). Complement components in normal pregnancy. Acta Pathol Microbiol Immunol Scand.

